# Management of dental disease in eared seals (family Otariidae): a Delphi approach

**DOI:** 10.3389/fvets.2025.1619326

**Published:** 2025-08-26

**Authors:** Claire A. Simeone, Nora Schwitzer, Shawn P. Johnson, José C. Almansa Ruiz, Yves Debosschere, Judy Force, Aaron Forsayeth, Jerzy P. Gawor, Margherita Gracis, Steven Holmstrom, Loïc Legendre, Lisa A. Mestrinho, Arlete R. Sogorb, Gerhard Steenkamp, Rebecca Tucker, Cedric Tutt, Ignacio Velázquez Urgel, Allison Woody

**Affiliations:** ^1^Sea Change Health, Sunnyvale, CA, United States; ^2^Conservation Department, Fundación Oceanogràfic de la Comunitat Valenciana, Valencia, Spain; ^3^Six Flags Discovery Kingdom, Vallejo, CA, United States; ^4^Veterinary Department, Dublin Zoo, Dublin, Ireland; ^5^MyVet Referral, MyVet Lucan, Dublin, Ireland; ^6^Bristol Vet Specialists, Bristol, United Kingdom; ^7^Veterinary Dental Services & Tsavran Consulting, Waregem, Belgium; ^8^Dentistry for Animals, Aptos, CA, United States; ^9^Advanced Animal Dentistry, Brisbane, QLD, Australia; ^10^Klinika Arka, Kraków, Poland; ^11^Clinica Veterinaria San Siro Anicura, Milan, Italy; ^12^Peter Emily International Veterinary Dental Foundation, San Pedro, CA, United States; ^13^West Coast Veterinary Dental Services Ltd., Vancouver, BC, Canada; ^14^Centre for Interdisciplinary Research in Animal Health (CIISA) and Associate Laboratory for Animal and Veterinary Sciences (AL4AnimalS), Faculty of Veterinary Medicine, University of Lisbon, Lisbon, Portugal; ^15^Research in Veterinary Medicine (I-MVET), Lisbon University Centre, Faculty of Veterinary Medicine, Lusófona University, Lisbon, Portugal; ^16^IPLUSO - Polytechnic Institute of Lusofonia, School of Health, Protection and Animal Welfare, Lisbon, Portugal; ^17^Centre for the Research and Technology of Agro-Environmental and Biological Sciences (CITAB- Inov4Agro), Faculty of Veterinary Medicine, University of Trás-os-Montes and Alto Douro (UTAD), Vila Real, Portugal; ^18^Department of Companion Animal Clinical Studies, Faculty of Veterinary Science, University of Pretoria, Pretoria, South Africa; ^19^Centre for Veterinary Wildlife Research, Faculty of Veterinary Science, University of Pretoria, Pretoria, South Africa; ^20^The Veterinary Dentist, Cape Town, South Africa; ^21^Odontovets, Barcelona, Spain; ^22^Advanced Zoological Dentistry, San Diego, CA, United States

**Keywords:** Otariidae, Delphi, best practices, dentistry, veterinary medicine, sea lion, fur seal

## Abstract

**Objective:**

Published literature is sparse on topics associated with eared seal (otariid) dentistry. The objective of this study was to establish consensus on effective management of dental disease in otariids, using a Delphi approach.

**Methods:**

A total of 25 veterinarians with experience managing dental disease in seven species of otariids participated in the Delphi process.

**Results:**

Oral lesions and their contributing risk factors were ranked according to perceived frequency. Consensus statements for best practices were agreed upon for a variety of topics within the categories of planning and preparation, procedural details, intervention strategies, and postoperative care. Panelist comments were collated into a Supplementary File to assist clinicians in forming their own conclusions on topics for which no consensus yet exists.

**Conclusion:**

Opportunities for future research include factors associated with oral lesions, ideal anesthetic management, identification of ideal candidates for endodontic therapies, ideal local and regional anesthesia, ideal suture and closure techniques, particularly with the goal of reducing dehiscence as a postoperative complication, and ideal postoperative care options.

## 1 Introduction

Otariids are eared seals, which include sea lions and fur seals. While otariid dentistry practices have been documented for decades (1), published literature is sparse and frequently limited to anatomical studies of skulls and teeth, or individual case reports (2–5). Many important clinical dental diseases do not lead to clinical trials or systematic data analysis. In these cases, clinicians and veterinary dentists use their experience and clinical judgement to guide their decision-making. Particularly in exotic and wildlife species, where case numbers are few and clinical experience is challenging to build, alternative methods are desired to guide treatment recommendations.

Given the lack of published data from clinical trials, which precludes a robust systematic review, consensus on how clinicians should approach dental disease in otariids can be gathered from a group of panelists with relevant experience using a Delphi approach. Delphi studies are used to combine clinical expertise and achieve consensus on preferred management approaches in human health research and specifically in dentistry (6, 7). The anonymity provided to participants by the Delphi method can minimize potential personal intimidation bias and produce more frequent and stable consensus when compared with other methods (8).

The objective of this study was to establish consensus on effective management of dental disease in otariids, using the Delphi method approach resulting in: (a) consensus statements for those topics for which general agreement exists; (b) a focus for discussion about where existing information from other species may be extrapolated for otariids; and (c) identification of future research priorities to provide data where gaps currently exist.

## 2 Materials and methods

### 2.1 Steering committee

A steering committee was formed consisting of four of the authors of the study (CAS, NS, SPJ, AW). The responsibility of the committee was to recruit panelists and to design, circulate and analyze the questionnaires. The steering committee made collective decisions regarding the methodology and data analysis.

### 2.2 Design

An electronic version of the Delphi method was used for the study (9). The study align with the Conducting and Reporting Delphi Studies (CREDES) recommendations (Supplementary File 1) to assure study rigor (10). A pilot survey was pre-tested by the members of the steering committee, modified, and then distributed to confirmed panelists. Panelist responses were blinded to all others, with only CAS having access to the unblinded data to allow for follow-up and coordination with panelists. Past studies have found that respondent fatigue has been observed to set in after two or three rounds (11) and extending rounds beyond two may not result in any dramatic advantage (12). Subsequent rounds are indicated until interquartile ranges are minimized and responses center around stable values (7, 13), and while three to four rounds are often indicated, studies that achieve end points after only two rounds are well documented in dentistry literature (14–17).

### 2.3 Panelists

Panelists were sought globally with a variety of veterinary qualifications. Experts that had a known publication record in the area of expertise were contacted directly via email, and a call for participation was published in both professional marine mammal and veterinary dental societies. Inclusion criteria included veterinarians with personal experience managing dental disease in an otariid species. Panelists were recruited over 8 weeks prior to the start of the study.

### 2.4 Delphi procedure

Questionnaires were hosted on Google Forms^1^, and panelists received an email containing a link to each of two questionnaires. Panelists had 6 weeks to complete each questionnaire, and two reminder emails were sent to those who had not yet completed the questionnaire. All panelists’ characteristics such as technical qualifications, number of dental procedures performed on otariids, and the otariid species with which they have experience were documented.

**Figure 1:**
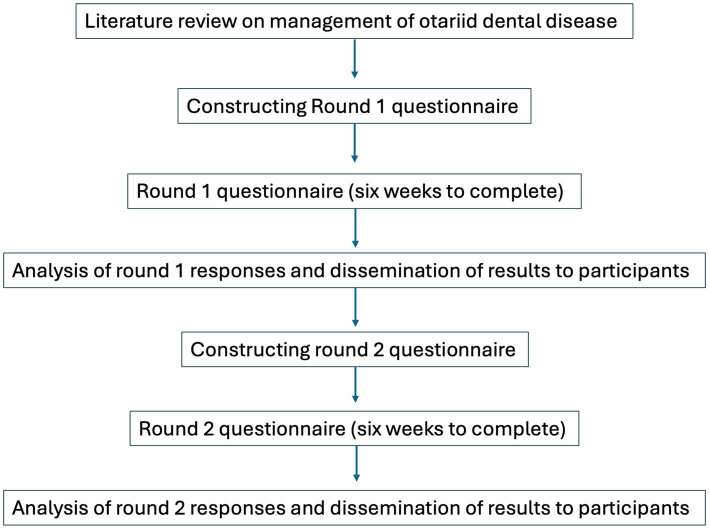
Procedure and timelines for panelists in the Delphi study.

Panelists were asked to provide their level of agreement or strength of recommendation for 20 questions, divided into the following five categories: (1) Oral Lesions; (2) Planning and Preparation; (3) Intervention Strategies; (4) Procedural Details; and (5) Postoperative Considerations. A five-point Likert scale (1 = strongest agreement or recommendation, 5 = weakest agreement or recommendation) was used throughout. “Not applicable” (N/A) was added as an option if the panelist did not have experience with the topic, which was analyzed as a 6 on the Likert scale. The consensus was assessed by analyzing descriptive statistics against pre-defined criteria for consensus. Open-ended follow-up was provided for each question to allow panelists to provide more detail and to add responses that may have been overlooked by the steering committee. The additional responses suggested by panelists were added to the round two questionnaire. If responses from round one questions reached pre-defined exclusion criteria they were excluded from the second round. Panelists were allowed 6 weeks to complete each round, and 4–6 weeks were taken for data analysis. Figure 1 describes the procedure and timeline of the study. The questionnaires can be found in Supplementary File 2, and an aggregate of all panelists’ comments can be found in Supplementary File 3.

### 2.5 Data collection and analysis

Results of the descriptive statistics were discussed with the steering committee before creating the second questionnaire. Descriptive statistics including median, interquartile range, and percentage of agreement were used to assess consensus in each round according to established criteria used in other Delphi studies (18, 19).

Criteria of consensus and exclusion were defined prior to the start of the study. Criteria of consensus included: median value of panelists’ Likert scale data </= 2; interquartile range (IQR) </= 1.0; percentage of agreement >70%. Criteria of exclusion included: median value of panelists’ Likert scale data >3; IQR >1.5; percentage of agreement <50%. A Likert scale is a rating scale used to measure opinions, attitudes, or behaviors. It consists of a statement or a question, followed by a series of five to seven answer statements. Respondents choose the option that best corresponds with how they feel about the statement or question. The interquartile range (IQR) is a measure of data spread. It is equal to the difference between the 75th and 25th percentiles, or the middle 50% of the data. In instances where stable disagreement existed that trended towards consensus against the statement, a consensus statement against the initial statement was proposed in the second questionnaire.

## 3 Results

### 3.1 Panelist demographics

A total of 42 potential panelists, either self-identified and contacted the steering committee, or were identified and contacted, were screened for participation. After assessment, a total of 25 panelists met the inclusion criteria and agreed to participate. Eighty percent of panelists were Diplomate veterinary dental specialists, 14% held another advanced veterinary dentistry degree or certification, and 8% were zoo veterinarians. Forty-four percent of panelists were based in North America, 36% in Europe, and 20% from Africa, Australia, and Asia. Panelists reported a variety of experience levels, with 36% reporting >10 dental procedures on otariids, 20% reporting 6–10 procedures, and 44% reporting 1–5 procedures. All 25 panelists (100%) completed two rounds of questionnaires. Panelists reported having experience working with seven species of otariids (Table 1).

**Table 1:** Otariid species with which panelists reported dentistry experience.

Common name	Scientific name
California sea lion	*Zalophus californianus*
Steller sea lion/northern sea lion	*Eumetopias jubatus*
South American sea lion	*Otaria byronia*
Australian sea lion	*Neophoca cinerea*
Northern fur seal	*Callorhinus ursinus*
South African fur seal/Cape fur seal/brown fur seal	*Arctocephalus pusillus*
Subantarctic fur seal	*Arctocephalus tropicalis*

Interquartile ranges were compared between panelists who had worked on <5 cases, and those with more experience with otariids. For responses to questions looking for consensus, 93% of answers had a difference in IQR of less than 2. For the two questions that diverged, more experienced panelists were more likely to strongly prefer to obtain their own intraoral radiographs during a procedure rather than relying on pre-procedure imaging (ΔIQR 2.75); and more experienced panelists were more likely to recommend an intervention for a case with malocclusion or gingival recession than colleagues with experience with fewer cases (ΔIQR 2). Consensus was ultimately achieved for both questions, suggesting that this divergence did not influence the outcome.

### 3.2 Oral lesions

The oral lesions observed in otariids were ranked according to the frequency perceived by the panelists (Table 2). The risk factors that were perceived to contribute to the observed lesions were ranked by the panelists according to their perceived association. Several lesions were added and assessed in the second questionnaire based on panelist comments from the first questionnaire - “tooth abrasion,” “mucosal ulceration,” “pulp granuloma,” “odontogenic cyst,” and “peripheral odontogenic fibroma”; no perceived risk factors were added in the second questionnaire.

**Table 2:** Oral lesions that have been observed in otariids, reported by panelists according to their perceived frequency.

Oral lesion	Median (Interquartile range)
Tooth fracture	1 (1–2)
Tooth abrasion*	1 (1–3)
Draining tract	2 (1–3)
Periodontal disease	3 (2–5)
Mucosal ulceration*	5 (3.5–6)
Soft tissue trauma*	5 (3–6)
Missing teeth	5 (3–6)
Pulp granuloma*	5.5 (2–6)
*Supernumerary teeth*	*6 (3–6)*
*Tooth resorption*	*6 (3.75–6)*
*Jaw fractures*	*6 (4.5–6)*
*Malformation*	*6 (5–6)*
*Neoplasia/cancer*	*6 (5–6)*
*Peripheral odontogenic fibroma*	*6 (5–6)*
Odontogenic cyst*	*6 (6–6)*

**Perceived risk factor**	**Median (Interquartile range)**
Chewing on foreign/environmental objects	1 (1.25–2.25)
Idiopathic/unknown cause	3 (1–6)
Restraint (squeeze cage, nets)	5 (2.25–6)
*Fighting (with conspecifics)*	*6 (3–6)*
*Work/performance-related injury*	*6 (5–6)*
*Self-mutilation*	*6 (5–6)*
*Early weaning*	*6 (6–6)*

Ranking ranges from 1 = most frequent to 5 = least frequent, and N/A = have not observed (treated as a ranking of 6). Starred (*) lesions were added and assessed in the second questionnaire based on panelist comments. Italicized lesions are considered rare, with most panelists reporting not observing the lesion or risk factor.

Panelists were asked to consider which clinical signs and/or lesions would cause them to recommend an intervention. Consensus was achieved for the lesions in Table 3. “Malocclusion causing trauma” did not achieve consensus, and three suggestions from panelists in the first round–“hemorrhage,” “nasal discharge,” and “sneezing” were included in the second round assessment but were also excluded due to low agreement.

**Table 3:** Oral lesions and clinical signs that achieved consensus among panelists that if observed, an intervention would be recommended.

Lesion	Median (Interquartile range)
Apical infection/draining tract	1 (1–1)
Facial swelling	1 (1–1)
Mass/growth	1 (1–1)
Shortened crown (e.g., abrasion, attrition, fracture, pulp exposure, etc.)	1 (1–1)
Mobile teeth	1 (1–2)
Inflammatory mucosa	2 (1.5–2)
Behavior changes	2 (1–2.25)
Gingival recession	2 (1.5–3)

Ranking ranges from 1 = strongest recommendation to 5 = weakest recommendation, and 6 = N/A, have not been observed.

### 3.3 Planning and preparation

Panelists refined a list of recommended steps to take when preparing for a procedure, which included “prepare equipment list,” “research specific anatomy,” “obtain pre-procedure radiographs,” “discuss expectations and plan with team members and staff,” “prepare backup equipment,” and “review patient chart/record.”

When asked how important it was to consult with dental specialists with experience working with an otariid species prior to their first procedure, consensus was achieved in the second round (median 1, IQR 1–2, 76% agreement).

When asked about financial constraints, the majority of panelists (55%) reported that financial constraints had been neither a delay or barrier to management of dental cases, while 31% reported financial constraints as delaying management and 14% reported financial constraints as a barrier to management.

Consensus was nearly unanimous that the presence of a veterinary anesthetist with experience working with the species was extremely important (median 1, IQR 1–1, 92% agreement).

Panelists were nearly evenly split (48% responding ‘yes’, 52% responding ‘no’) on whether a maximum procedure time should be established and adhered to. Given the large divide, questionnaire two worked to achieve consensus on the following statement: “There is no consensus on whether a specific maximum procedure time should be established. Discussing all the factors that impact procedure time with team members beforehand is important to formulate a realistic plan.”

Panelists had a wide range of recommendations regarding pre-procedure imaging. Specific imaging recommendations did not achieve consensus, but consensus was achieved on the following best practices statement: “Given the challenges of performing high quality diagnostic radiographs, we welcome pre-procedure imaging, but dental practitioners will generally obtain their own intraoral radiographs prior to performing work.”

Near unanimous consensus was achieved against recommending a separate anesthetized procedure to perform pre-procedure radiographs. Consensus was achieved on the following best practices statement: “A separate anesthesia is typically not recommended because dentists will perform their own radiographs during the procedure.”

Several imaging practices were not carried forward due to low agreement, including “voluntary (awake) intraoral radiographs,” “voluntary (awake) skull radiographs,” and “CT/CBCT.”

### 3.4 Intervention strategies

Consensus was achieved for “extraction” as a technique for addressing non-vital or pulp-exposed teeth (median 1, IQR 1–1, 92% agreement), and for teeth affected by moderate to advanced periodontal disease (median 1, IQR 1–2, 80% agreement). Recommendations for “endodontic therapies” did not reach consensus in the first questionnaire (median 4.5, IQR 3–5, 21% agreement). Consensus was achieved in the second questionnaire with the statement: “Endodontics should only be used in select cases where the following criteria are met: closed apex, lack of resorption, and where radiographic follow-up is possible with the animal” (median 1, IQR 1–1.25, 79% agreement).

“Periodontal therapies” did not reach consensus in the first questionnaire (median 3.5, IQR 3–6, 21% agreement). In the second questionnaire, clarification was sought by evaluating two types of periodontal therapies. Recommendations for “minor periodontal therapies (such as debriding of pockets)” did not reach consensus (median 2, IQR 1–3, 62% agreement) but arrived close to the defined cutoff. Recommendations for “advanced periodontal therapies (such as guided tissue regeneration or sliding tissue flap)” were not carried forward due to low agreement.

### 3.5 Procedural details

If many teeth are affected, the panelists reached a consensus on approaching the procedure by addressing the most severe issues first, and staging procedures until complete (triage plus staging) (median 1, IQR 1–1, 88% agreement). Low agreement was found with staging only, triage only, or addressing all issues at once; these options were omitted from the second questionnaire.

In the first questionnaire, discussion of local anesthetics led to consensus that both the “local block site” and “local anesthetic dose” were important to consider, and consensus was nearly complete with “local anesthetic drug” (median 1, IQR 1–2.75, 73% agreement). In addition to requesting details on these factors from panelists in the second questionnaire, consensus was achieved on the following statement: “A local or regional block is recommended when performing a procedure likely to cause postoperative pain.”

Consensus was achieved in the first questionnaire that “suture material” and “suture size” are important factors to consider when closing an extraction site, and consensus was nearly achieved for “closure pattern” (median 1.5, IQR 1–2.75, 73% agreement). In the second questionnaire, details were requested from the panelists about their recommendations on suture.

There was low agreement in the first questionnaire for all statements about antibiotic use. In the second questionnaire unanimous consensus was achieved for the following statement: “In general, prophylactic antibiotic use is not recommended for dental procedures. Judicious antibiotic use may be needed in cases that warrant it” (median 1, IQR 1–1, 100% agreement).

When asked whether bone graft is recommended there was low agreement (median 6, IQR 6–6, 4% agreement). The majority of respondents (64%) have not used bone graft in otariid dental cases, and 16% would specifically recommend against its use.

Specific comments that panelists made regarding their recommendations for procedural details can be found in Supplementary File 3.

### 3.6 Postoperative considerations

**Figure 2:**
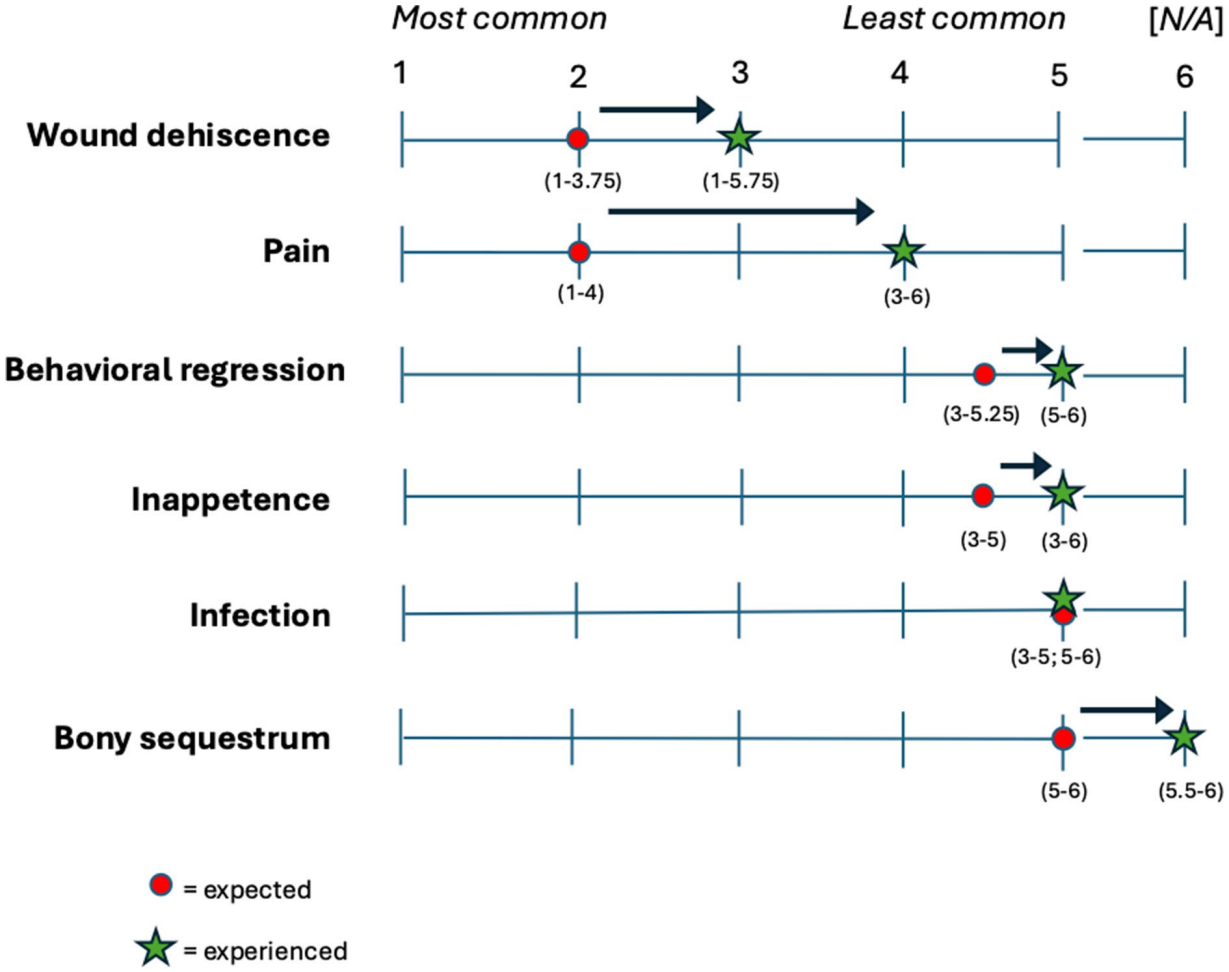
Expected (circle) and experienced (star) complications reported by panelists according to their perceived frequency. Ranking ranges from 1 = most frequent to 5 = least frequent, and 6 = N/A, have not observed. Interquartile range (IQR) is reported in parentheses.

When asked about what complications they expected to encounter when planning for a procedure, and then what complications they actually experienced after the procedure, panelists reported the complications described in Figure 2.

When considering a successful treatment, panelists achieved consensus that the most clinically relevant outcomes were “resolution of pain,” “resolution of inflammation/infection,” “jaw stability,” and “function.” “Client satisfaction” and “aesthetics” were not carried forward due to low agreement.

When an animal is recovering from an intervention, panelists achieved consensus that the postoperative care factors “reducing access to toys/cribbing objects,” “analgesia,” and “photodocumentation” were most important for a positive outcome. Several factors were not carried forward due to low agreement, including “restricting water access,” “oral rinses,” and “laser therapy.” Important clinically relevant outcomes and postoperative care factors are reported in Table 4.

**Table 4:** Clinically relevant outcomes and postoperative care factors that were perceived to be important for a positive postoperative outcome.

**Clinically relevant outcomes**	**Median (Interquartile ranges)**
Pain (resolution)	1 (1–1)
Inflammation/infection (resolution)	1 (1–1)
Jaw stability	1 (1–1)
Function*	1 (1–1)

**Postoperative care factors**	
Restricting access to toys/cribbing objects	1 (1–1)
Analgesia	1 (1–1)
Photodocumentation*	1 (1–2.75)

These factors achieved consensus during the Delphi process. Starred (*) lesions required discussion in the second questionnaire to achieve consensus. Ranking ranges from 1 = most important to 5 = least important, and N/A = would not recommend this postoperatively (calculated as 6 on the Likert scale).

During recovery, the recommended postoperative or follow-up surveillance that achieved consensus among panelists was “visual observation by keepers or trainers” (median 1, IQR 1–1, 96% agreement), and photodocumentation (median 1, IQR 1–1, 88% agreement). Several postoperative surveillance factors were not carried forward due to low agreement, including “visual observation by veterinarians,” “visual observation by veterinary dentists,” and “restrained/anesthetized follow-up.” There was consensus against anesthetized postprocedure follow-up (median 5, IQR 4–5.25, 84% *dis*agreement). The authors believe a statement, “during the healing period, a separate anesthesia for follow-up is typically not necessary” would have achieved consensus but elected against a third questionnaire for this single outcome.

All consensus statements from the Delphi process are summarized in Table 5.

**Table 5:** Consensus statements agreed upon by panelists during the Delphi process.

Category	Topic	Consensus statement
*Planning and preparation*	Preparation steps	When planning for a procedure, a list of suggested steps to prepare include: -Prepare an equipment list -Research specific anatomy -Discuss expectations and plan with team members and staff -Obtain pre-procedure radiographs -Prepare backup equipment -Review patient chart/record
	Pre-procedure imaging	Given the challenge of performing high quality, diagnostic radiographs, pre-procedure imaging is welcomed, but dentists will generally obtain their own intraoral radiographs before performing work.
		A separate anesthesia is typically not recommended for pre-procedure imaging because dentists will perform their own radiographs during the procedure.
*Procedural details*	Anesthesia	Working with a veterinary anesthetist with experience with the species is extremely important.
	Maximum procedure time	There is no consensus on whether a specific maximum procedure time should be established. Discussing all factors that impact procedure time with team members beforehand, and throughout the procedure is important to formulate a realistic plan.
	Local anesthesia	A local or regional anesthetic block is recommended when performing a procedure likely to cause postoperative pain.
	Antibiotic use	In general, prophylactic antibiotic use is not recommended for dental procedures. Judicious antibiotic use may be needed for specific cases that warrant it.
*Intervention strategies*	Extraction	If a non-vital or pulp-exposed tooth requires intervention, extraction would be recommended.
		If a moderate to advanced periodontal disease-affected tooth requires intervention, extraction would be recommended.
	Endodontics	Endodontics should only be used in select cases where the following criteria are met: closed apex, lack of resorption, and where radiographic follow-up is possible with the animal.
	Staging	If many teeth are affected, the most severe issues should be addressed first, and additional procedures would be staged until complete.
*Postoperative care*	Follow-up surveillance	During recovery, visual observation by keepers/trainers, and photodocumentation are recommended.
		During the healing period, a separate anesthesia for follow-up is typically not necessary.

## 4 Discussion

### 4.1 Oral lesions

The lesions reported as most frequent in this study (abrasion, fracture, draining tracts, and periodontal disease) are similar to what has been reported in the literature. Among wild populations of California sea lions and Steller sea lions, common dental conditions include tooth wear (attrition, abrasion), and periodontitis, with adult males more frequently affected than females (3). Teeth with pulp exposure have also been reported in many captive populations and appear to be a common feature in young adult sea lions (3, 20, 21).

While chewing on foreign/environmental objects was the most common factor suspected in many oral lesions, idiopathic or unknown causes remain common. Further investigation into the frequency of various factors and their associations with oral lesions may be warranted in collaboration with husbandry staff who have more continuous interaction with and observation of the animals.

When asked to consider which clinical signs and/or lesions would cause the panelists to recommend an intervention, consensus was not achieved in the first round for malocclusion. The steering committee felt that further information might be needed to clarify that the question was not regarding aesthetics, so in the second round panelists were asked to consider the presentation of an animal which had a malocclusion that was causing trauma. Consensus was still not achieved. The authors found this surprising as malocclusion is known to cause tooth wear and mucosal trauma, among other problems (22), and panelists achieved consensus for recommending interventions for both shortened crowns and inflammatory mucosa.

One limitation of this study is that it did not differentiate between animals housed in managed care (e.g., zoos and aquaria) and those undergoing short-term rehabilitation for release. As the reported lesions and treatments reflect the collective opinions of experienced panelists rather than systematically gathered clinical data, the findings do not account for species-specific differences or context-specific factors (such as long-term captivity versus short-term rehabilitation), which may significantly influence both pathology and treatment decisions. In addition, data were not collected on specific numbers of animals treated from each species, as case numbers were likely too small to draw any meaningful comparisons between species. Future study is warranted to determine species-specific differences in diagnosis and management of dental disease in different otariid species.

### 4.2 Planning and preparation

#### 4.2.1 Colleague consultation

Consensus was achieved in the first round with near unanimous agreement that involving a veterinary anesthetist with experience working with the species was extremely important. While true anesthetic risk is unknown for the majority of marine mammal species, it is reportedly higher than domestic species due to a variety of factors (23). However, consensus was not achieved on whether it was important to consult with dental colleagues with experience working with otariids prior to their first procedure. Comments were mixed, with some panelists remarking that prior consultation was extremely valuable, while others remarked that few experts exist in the treatment of dental conditions in this species, and that they were pioneers that did not have literature or others to rely on. Given that the majority of panelists agreed that asking for advice prior to a new procedure was important, the hope is that collaborative efforts such as this study will make it easier to find that information in the future.

#### 4.2.2 Pre-procedure imaging

Veterinary practitioners depend on imaging techniques as a critical component of comprehensive oral health assessment. Imaging allows noninvasive examination, facilitating the diagnosis and evaluation of dental pathology within the oral cavity. Intra-oral radiography remains the primary imaging method in veterinary dentistry. Currently, there is no published data on the prevalence of dental disease in otariid populations, though it is widely accepted that oral examinations are incomplete without imaging.

According to WSAVA guidelines (24), anesthesia-free dentistry practices are discouraged for canine and feline patients as they may compromise patient welfare, increase practitioner risk, and limit diagnostic accuracy, particularly for radiographic assessment. Most panelists extended these same recommendations to otariids. However, some argued that given the challenges of anesthesia in these species, high-quality intraoral pre-procedure radiographs can be obtained with a trained, cooperative, awake animal in some situations. Panelists were able to achieve consensus on two statements: first, welcoming pre-procedure imaging if it exists, and second, that a separate anesthesia is typically not recommended for pre-procedure imaging alone, because dental practitioners will generally obtain their own intraoral radiographs prior to performing work.

An alternative imaging modality, computed tomography (CT), offers a faster diagnostic option with potential advantages for multi-rooted maxillary teeth in dogs and cats. CT imaging eliminates root and bone structure superimposition and avoids positional distortions, as well as provides a more accurate assessment of alveolar margin height (25). In canine and feline patients, a CT slice thickness of 0.5–1 mm is generally sufficient to replace dental radiography (26). For otariid species, CT may be especially valuable, offering practitioners detailed visualization of tooth root morphology and alveolar bone architecture, which can make the planning of more complex procedures like mandibular canine extraction easier. Consensus was not achieved about CT as a preferred imaging modality, with comments split between the superior visualization that CT provides for certain cases versus the limited access that many clinics currently have to CT. Digital Tomosynthesis (DT) is a type of 3D imaging modality that is currently being investigated by veterinarians as a means of evaluating dental structures without superimposition (27), but at a lower investment cost and smaller footprint than CT. It was not discussed in this study but could be considered in the future.

### 4.3 Intervention strategies

#### 4.3.1 Endodontics

Endodontic treatment facilitates the retention of teeth affected by pulpal or periapical pathology (28). Root canal therapy and vital pulp therapy are the most commonly performed endodontic procedures in veterinary dental practice. The therapies provide minimally invasive alternatives to extraction, effectively preserving tooth structure and function while demonstrating a high success rate in dogs (29–33).

The essential steps of root canal therapy include disinfection, shaping, and obturation of the root canal system. Otariid teeth present challenges in achieving these steps. The root walls of immature sea lion canine teeth are significantly divergent apically and the apexes remain open for an extended period of time. Given normal physiological influences on tooth development (34), the authors have experienced that sea lion canine tooth apexes will typically be open for the first 10 years of life. Captive sea lions often are presented for pulp exposed teeth before 5 years of age. Consequently, the anatomy and development of the teeth combined with the challenges of adequate postoperative follow up under general anesthesia make proper root canal treatment difficult to achieve, and good root canal candidates nearly impossible to find.

In a recent study examining seven teeth from two California sea lions housed in captivity, Nemec et al. found pulp exposure in two teeth with no radiographic evidence of endodontal disease (21). Histologically, the teeth were viable, showing only coronal pulpitis and pulpal polyp formation, suggesting that vital pulp therapy could theoretically be a viable alternative to tooth extraction in sea lions.

There was agreement among all panelists in our survey that endodontal disease warrants extraction of the diseased teeth. There seemed to be a general desire to preserve the teeth of sea lions, particularly the canines, but no positive endodontic treatment outcomes were reported in the survey. Ultimately, the group did not achieve consensus on a revised statement regarding endodontic treatments in round two of the questionnaire. In order to achieve future consensus, ideal endodontic treatment candidates must be identified, and successful cases need to be documented and described in the literature.

#### 4.3.2 Periodontal disease

Periodontal disease is the inflammation and infection of the soft tissues and bone holding the tooth in place. It results in the progressive loss of attachment and is classified in veterinary medicine using the American Veterinary Dental College (AVDC) stages of periodontal disease index (35). The prevalence of periodontal disease in California sea lions has been documented at around 20% in museum skull specimens (2). No prevalence studies on live populations have been done to date.

The black coating on sea lion teeth should not be confused with dental calculus and is generally adjacent to healthy gingiva. The staining is believed to be the result of chromogenic bacteria that gather on the enamel and are not pathogenic (20). True dental calculus accumulation is beige to tan as in other species and an uncommon finding in otariids.

Periodontal therapy is an umbrella term for all surgical and non-surgical treatments used to stop the progressive loss of tooth attachment and restore gingival health. Panelists in this study achieved consensus for recommending extraction as a treatment for teeth affected by moderate (stage 3) to severe (stage 4) periodontal disease. Consensus was not achieved for recommending minor periodontal treatments such as debriding of periodontal pockets (closed root planing) or use of a subgingival pharmacotherapeutic agent. Poor agreement was found among panelists for advanced periodontal treatments such as guided tissue regeneration or sliding tissue flaps. Given that dehiscence was reported to be the most observed postoperative complication in otariids, it is likely to be a barrier to the success of more advanced periodontal therapies until it can be more effectively prevented.

#### 4.3.3 Staging

The decision to stage surgical procedures or perform them in a single session depends on several factors, including the complexity of the surgery, patient health, recovery needs, and access to experts. Staging procedures can reduce individual procedure time under anesthesia, reduce operator fatigue, and allow for patient recovery between stages. On the other hand, single procedures may be more convenient for patients/facilities, reduce total anesthesia time, and reduce overall procedure cost.

Panelists in this study reached consensus on approaching complex cases by addressing the most severe issues first and staging remaining treatments until complete. However, only half of the panelists believed that setting a pre-determined “cut-off time” for procedures was practical or beneficial. Of those that recommended establishing a maximum procedure time, responses varied widely, with suggested time frames ranging from 45 min to 4 h. Several panelists highlighted the lack of specific scientific guidance on safe anesthesia durations for otariids and emphasized that the decision should depend on the patient’s stability under general anesthesia. Evidence from both human and veterinary literature suggest that procedure length may influence procedure outcome, although both present mixed opinions on this topic (36–42). Limited studies in otariids suggest that other factors, such as health status or anesthetic drug combination used, may have a greater association with perianesthetic mortality than duration of anesthetic period (43, 44). There is an opportunity for future research to better characterize the association between anesthetic risk and treatment complications with increasing anesthesia times for otariid species.

### 4.4 Procedural details

The conical taper anatomy of otariid canine teeth, especially sea lions, present significant challenges for extraction. However, surgical extraction is a must in most cases, with the wide flaring apex of immature canine teeth precluding root canal therapy. Canine tooth extraction is a step-by-step procedure that involves creating a mucogingival flap and performing a wide ostectomy or alveolectomy on the buccal aspect of the tooth (1, 21). Once sufficient alveolectomy has been achieved, the tooth is luxated and elevated until it can be removed atraumatically with extraction forceps. The alveolus is then gently debrided, and the mucogingival flap is closed using an absorbable monofilament suture.

While most panelists in this study agreed with the above open extraction technique, one suggested that closed extractions could be performed successfully with precise luxation techniques. Several panelists noted that canine tooth extractions often necessitate the removal of the first and second single-rooted premolar teeth to facilitate the procedure. In all cases, it is not known what level of canine tooth maturity the commenters have had experience extracting and if that played a role. Panelists reported using various dental bur types during extraction, depending on personal preference, including round carbide burs, crosscut tapered fissure burs, and specialized root tip burs. Some panelists also employed automated periotomes and magnetic osteotomes.

Despite one panelist reporting good results with placement of bone grafts, there was general agreement against their use in extraction sites. Concerns about an increased risk of infection, loss of costly product if dehiscence occurred, and general lack of need were cited as reasons against the use of bone graft materials.

Suture material size for the mucogingival flap closure ranged from 3/0 to 5/0 and the panelists disagreed on the optimal suture size that would best align with the natural strength of the tissue. Needle types (cutting or tapered) and suture patterns (single interrupted, cruciate, horizontal mattress or simple continuous) also differed based on personal preference. Many panelists reported that the tough, non-elastic gingiva of California sea lions made tension-free, precise apposition of the wound edges challenging. Flap dehiscence was the most frequently reported postoperative complication. Dehiscence of other surgical sites such as skin incisions have been documented in otariids, and recommended preventative measures include the use of tension-relieving suture patterns and the avoidance of poorly vascularized tissue (45, 46). The need for wide bone excision during extractions, as noted by several panelists, may contribute to increased local tissue tension. Further research is warranted to clarify contributing factors.

The British Small Animal Veterinary Association (BSAVA) and American Veterinary Medical Association (AVMA) guidelines for responsible antibiotic use emphasize several key principles: prescribe antibiotics only when necessary, explore alternative treatments where possible, use an optimized dosage protocol for effective treatment, perform cytology and culture before prescribing, and follow established categorizations of antibiotics (47–49). Panelists crafted a consensus statement against prophylactic antibiotic use that aligns with these recommendations, noting that in some cases judicious antibiotic use may be warranted.

Panelists achieved consensus that a local or regional anesthetic block is recommended for a procedure that is likely to cause pain. However, there was a wide variety of suggestions among local anesthetic drugs and types of anesthetic block employed. No published studies exist on this topic for otariids.

### 4.5 Postoperative considerations

Regarding postoperative care, panelists achieved consensus that factors important to a positive outcome include proper analgesia, observation by keepers/trainers, photodocumentation of surgical sites, and limiting access to toys or cribbing objects. Beyond these factors there was low agreement, with suggestions and comments about variable postoperative management techniques.

Readers are directed to Supplementary File 3 to review panelist comments and recommendations. Many opportunities exist for future research including surgical techniques, suture size, patterns, and techniques, local/regional anesthesia, and factors in postoperative management.

## 5 Conclusion

Soliciting the input of a group with varied experience with the topic at hand has limitations, namely that the results remain a collection of opinions. Given that consensus was achieved on nearly every topic after two rounds the steering committee decided against pursing additional rounds, although additional rounds could have influenced the findings. The use of the Delphi method can be most useful where concrete data do not exist (50). We believe the information gathered from current panelists in this study will help improve and advance dentistry in otariids and identify areas where research is needed to better characterize the best practices for dentistry in otariids. In particular, this study identified the following topics as opportunities for future research: (1) the various factors associated with the cause of oral lesions, (2) ideal anesthetic management, (3) identification of ideal candidates for endodontic therapies, (4) ideal local and regional anesthetic drugs and approaches, (5) ideal suture and closure techniques, particularly with the goal of reducing dehiscence as a postoperative complication, and (6) ideal postoperative care options, including analgesic drug combinations and durations.

## Data Availability

The original contributions presented in the study are included in the article/Supplementary material, further inquiries can be directed to the corresponding author/s.

## Footnotes

^1^ www.forms.google.com
